# The Role of Lipoproteins in Mycoplasma-Mediated Immunomodulation

**DOI:** 10.3389/fmicb.2018.01682

**Published:** 2018-07-31

**Authors:** Alexei Christodoulides, Neha Gupta, Vahe Yacoubian, Neil Maithel, Jordan Parker, Theodoros Kelesidis

**Affiliations:** David Geffen School of Medicine, University of California, Los Angeles, Los Angeles, CA, United States

**Keywords:** mycoplasma, lipoproteins, immune system, immune modulation, inflammation

## Abstract

Mycoplasma infections, such as walking pneumonia or pelvic inflammatory diseases, are a major threat to public health. Despite their relatively small physical and genomic size, mycoplasmas are known to elicit strong host immune responses, generally inflammatory, while also being able to evade the immune system. The mycoplasma membrane is composed of approximately two-thirds protein and one-third lipid and contains several lipoproteins that are known to regulate host immune responses. Herein, the immunomodulatory effects of mycoplasma lipoproteins are reviewed. A better understanding of the immunomodulatory effects, both activating and evasive, of Mycoplasma surface lipoproteins will contribute to understanding mechanisms potentially relevant to mycoplasma disease vaccine development and treatment.

## Introduction

Mycoplasmas cause a wide variety of human disease, such as asthma, bronchiectasis, and pelvic inflammatory diseases (Metaxas et al., 2015). Several species of mycoplasma such as *Mycoplasma pneumoniae* in the respiratory tract, *M. genitalium* in the genitourinary tract cause human disease (Metaxas et al., 2015). Mycoplasma infections can place a big burden on healthcare systems, with more than 100,000 annual hospitalizations arising from *M. pneumoniae* in the US alone (Atkinson et al., 2008). In addition, mycoplasma infections of the respiratory and urogenital tract can be chronic. A better understanding of the pathogenesis of mycoplasma infections will contribute to vaccine development and treatment.

A key characteristic of the pathogenesis of chronic mycoplasma infections is the cross-talk between mycoplasmas and the host immune system (Atkinson et al., 2008). An inflammation response induced by the host’s immunity is one of the main characteristics of *M. pneumoniae* infection and contributes to clinical presentations. Elucidating the mycoplasma cellular components will help us understand how the relatively small, and biochemically simple, mycoplasmas are capable of eliciting strong and chronic immune responses (Himmelreich et al., 1996). Mycoplasmas can induce proinflammatory response through secreted toxins, surface antigens and other unclear mechanisms (Yeh et al., 2016). Considering that Mycoplasma lack cell walls, cell wall proteins do not interacting with the host’s immune system (Pereyre et al., 2016). Analysis of the mycoplasmal cell membrane demonstrated that several lipoprotein surface antigens elicit strong immune responses (Muhlradt et al., 1997) through a distinct pathway than that seen with lipopolysaccharides (Rawadi and Roman-Roman, 1996). Unlike numerous other bacterial species, a large portion of mycoplasmal genome (6.68%) is dedicated to encoding various lipoproteins (Dandekar et al., 2000; Hallamaa et al., 2006). The functionality of these genes is unknown, but they may drive adhesion and/or the initial stages of mycoplasmal infection since their products are cell surface proteins. Certain mycoplasmal lipoproteins have exonuclease activity that may be involved in the function of the ATP-binding cassette (ABC) transport system to import nucleic acid precursors (Schmidt et al., 2007). Little is known about the transcriptional regulation that occurs within these genes, especially since the mycoplasmal genome lacks many of the transcriptional regulators found in other bacteria (Himmelreich et al., 1996; Dandekar et al., 2000).

The lipoylation mechanism of lipoproteins in mycoplasmas is similar to that of other bacteria (Rottem, 2003). Bacterial lipoproteins play a major role in the cross-talk between bacteria and host immune responses. Bacterial lipoproteins contain a lipoylated amino-terminal cysteinyl residue that is usually *N*-acylated (Braun and Hantke, 1974). However, an *N*-acyltransferase gene has not been found in *M. pneumoniae* or *M. genitalium* genome (Himmelreich et al., 1996) and many mycoplasmal lipoproteins are not *N*-acylated [such as lipoproteins from *M. gallisepticum* (Jan et al., 1996; Muhlradt et al., 1997)]. Triacylation or diacylation of lipoproteins affects their recognition from different Toll like receptors (Jan et al., 1995). Thus, the unique structure of mycoplasmal lipoproteins can determine their immunomodulatory properties. Mycoplasma lipoproteins are all known to induce expression of proinflammatory cytokines including IL-1β, IL-6, and IL-2 (Garcia et al., 1998; Hoek et al., 2005). Several lipoproteins have been identified in mycoplasmas (**Table 1**).

**Table 1:** Major lipoproteins in mycoplasma species (isolated in humans).

Lipoprotein/Gene ID	Species of Mycoplasma	Original function	Immunomodulatory effect	References
MPN052, MPN162, MPN415, MPN602, MPN611	*M. pneumoniae*	F_0_F_1_ ATP Synthase subunit b	NF-kB activation, Pro-inflammatory diacylated lipoprotein	Shimizu et al., 2008, 2014
MPN597	*M. pneumoniae*	F_0_F_1_ ATP Synthase subunit 𝜀	Induction of inflammation, activation of the autophagy/TLR4-mediated pathway	Shimizu et al., 2014; Shimizu, 2016
MPN611 MPN162 MG-142 MG-149	*M. pneumoniae M. pneumoniae M. genitalium M. genitalium*		Activate NF-κB through TLR pathways	Shimizu et al., 2007
MPN372	*M. pneumoniae*	ADP-ribosylating toxin, CARDS toxin	Induces extensive vacuolization and ultimate cell death of mammalian cells, facilitates adhesion to human surfactant protein A (hSP-A), activation of inflammasome	Kannan et al., 2005; Kannan and Baseman, 2006
MPN333	*M. pneumoniae*	ABC Transporter	Activation of the autophagy/TLR4-mediated pathway	Shimizu, 2016
MPN142, MPN447, MPN453 Lp44	*M. pneumoniae M. salivarium*	Cytadherence	Activation of inflammasome	Shibata et al., 2000, p. 2; Waldo and Krause, 2006; Spuesens et al., 2011; Balish, 2014; Widjaja et al., 2015
MPN141	*M. pneumoniae*	Cytadherence, P1 adhesin	Induction of pro-inflammatory cytokine production	Waldo and Krause, 2006; Kannan et al., 2011

**FIGURE 1:**
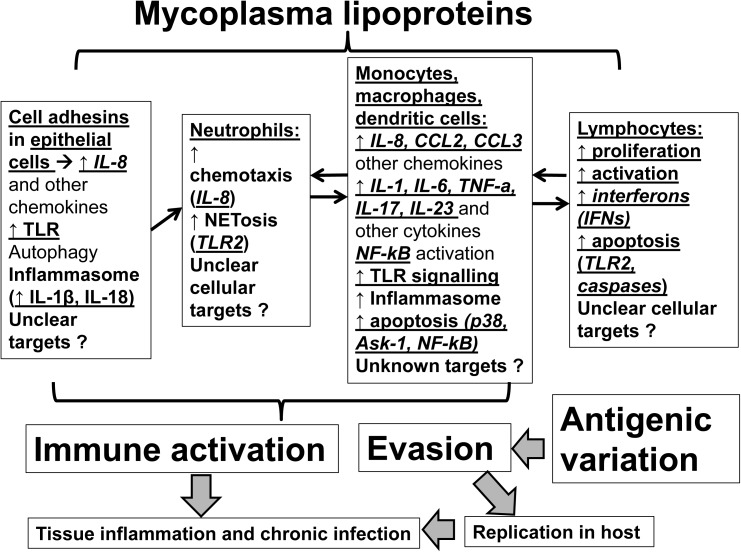
Mechanisms of actions of mycoplasmal lipoproteins: Mycoplasmal lipoproteins lead to inflammatory responses and immune system activation through direct effects on both epithelial and immune cells. Initial adhesion to epithelia of respiratory tract is mediated through adhesins such as P1 (MPN141) as well as other accessory proteins. Epithelial cell activation as well as mycoplasmal lipoproteins induce secretion of chemokines such as IL-8 that promote neutrophilic infiltration and activation of neutrophils (through several mechanisms including NETosis). Neutrophilic activation is followed by activation of myeloid cells (monocytes, macrophages, dendritic cells) that ultimately leads to activation of adaptive immunity (lymphocytes). A key family of receptors that play an important role in recognizing, and inducing responses to Mycoplasmal lipoproteins are the Toll-Like Receptors (TLRs such as TLR2 and TLR6) that are key players in inflammatory responses upon infection. Mycoplasmal lipoproteins can also activate cells through the inflammasome and the pre-cursors of proinflammatory cytokines, such as IL-1β and IL-18, can be cleaved and activated in a caspase-dependent manner prior to their release. Non-TLR or inflammasome dependent mechanisms by which Mycoplasmal lipoproteins can lead to inflammatory responses and immune activation also exist (such as autophagy). TLRs (such as TLR-2 and TLR-6), also play an important role in inducing apoptosis in both monocyte and lymphocyte cell lines through induction of p38 MAPK, the apoptosis signal-regulating kinase (ASK1), caspase and the NF-kB pathways and the release of Nitric Oxide from macrophages. See text for further details. Given poorly understood mechanisms, further studies are needed to elucidate the mechanisms of immunomodulation mediated by mycoplasmal lipoproteins.

Although Mycoplasma lipoproteins may function as immune-activators within their host, they also regulate mycoplasma colonization and translocation across mucosal membranes and facilitate host immune evasion (Citti et al., 1997; Chambaud et al., 1999). Understanding lipoprotein-induced immunomodulation will aid in developing novel treatments against Mycoplasmas (Martinez de Tejada et al., 2015). Herein, we review the evidence on the effects of mycoplasmal lipoproteins on host immunity (**Figure 1**). Given the complex immunomodulatory effects of mycoplasma lipoproteins we will dissect these effects by specific cell type (epithelial cells, neutrophils, myeloid cells, lymphocytes).

### Effects of Mycoplasmal Lipoproteins on Epithelial Cells

Epithelial cells are the main targets of *M. pneumoniae* and secrete cytokines in response to infections (Fahey et al., 2005). Mycoplasmas directly induce *in vitro* production of chemotactic cytokines such as IL-8 by bronchial epithelial cells (Chambaud et al., 1999; Peng et al., 2007; Chen et al., 2016). Mycoplasmal lipoproteins may also have a major role in regulation of the cytokine response in respiratory epithelial cells during *M. pneumoniae* infection (Eckmann et al., 1995; Yang et al., 2002). Similar to the role of respiratory epithelial cells in eliciting a response during *M. pneumoniae* infection, epithelial cells lining the vagina and cervix may induce pro-inflammatory cytokines upon exposure to lipoproteins present in *M. genitalium* (McGowin et al., 2009). However, the exact mechanisms that contribute to differential effects of mycoplasma lipoproteins on epithelial compared to immune cells remain unclear. Epithelial cells are also able to have an effect on Mycoplasmas. Upon contact with human epithelial cells *in-vivo, M. pneumoniae* can undergo transcriptional changes in lipoprotein related genes (Hallamaa et al., 2008). Such an alteration in the genetic profile of the Mycoplasma upon coming into contact with epithelial cells represents the two-way dynamic existing between Mycoplasma and the host, where both parties are able to influence each other. Thus, the initial release of proinflammatory cytokines and chemokines by epithelial cells infected by Mycoplasmas can also induce early recruitment of immune cells such as neutrophils.

### Effects of Mycoplasmal Lipoproteins on Neutrophils

Mycoplasma respiratory infections lead to the recruitment of polymorphonuclear leukocytes and subsequently monocytes/macrophages (M/M) and lymphocytes (Kruger and Baier, 1997). The attraction of leukocytes to the site of infection is controlled by chemokines, which are chemotactic cytokines. The *M. pneumoniae*/IL-8/neutrophil axis likely plays a vital role in the pathogenesis of MPP and mycoplasmas directly induce *in vitro* production of chemotactic cytokines such as IL-8 by bronchial epithelial cells (Chambaud et al., 1999; Peng et al., 2007; Chen et al., 2016). An *M. fermentans* lipoprotein fraction induces secretion of IL-8 from cultured human M/M (Chambaud et al., 1999; Peng et al., 2007; Chen et al., 2016). The expression of such chemokines allows for the recruitment of leukocytes, such as neutrophils, to the site of infection, and it is this influx of leukocytes that comes to partially contribute to the inflammatory response seen upon mycoplasma infection (Wu et al., 2007).

Except for IL-8 and neutrophil chemotaxis, mycoplasma lipoproteins also induce NETosis. Neutrophil extracellular traps (NETs) are a major component of the first line of defense against invading pathogens and they involve a pathogen facilitated cell death by which neutrophils can extrude chromatin structures loaded with anti-microbial molecules to eliminate pathogens (Gomez-Lopez et al., 2017). Experiments using *M. agalactiae* have shown that mycoplasmas are capable of inducing NETosis upon infection, and mycoplasma lipoproteins are the component primarily responsible for activation of these pathways in neutrophils (Cacciotto et al., 2016). Furthermore, it has also been shown that lipoproteins induce Toll-like Receptor 2 (TLR2) signaling that plays a pivotal role in neutrophil NETosis (Cacciotto et al., 2016; Xu et al., 2017). Notably, NET formation ability decreases with patient age, and this may contribute to the susceptibility of older patients to mycoplasmal pathogens such as *M. pneumoniae* (Xu et al., 2017). More research is needed on the ability of lipoproteins to induce NETosis. Thus, mycoplasmal lipoproteins regulate neutrophilic host immune responses, which are the mainstay of pathogenesis of mycoplasmal infections. These early neutrophilic responses can also contribute to inflammatory responses from myeloid cells.

### Effects of Mycoplasmal Lipoproteins on Myeloid Cells

Mycoplasmas induce activation of cytolytic activity of macrophages, and stimulation of cytokines (interleukin [IL]-1, IL-6, tumor necrosis factor-alpha [TNF-α]). Although several mycoplasmal proteins can induce these proinflammatory responses (Chambaud et al., 1999; Peng et al., 2007; Chen et al., 2016), mycoplasmal lipoproteins seem to be a major instigator of production of proinflammatory cytokines by myeloid cells such as M/M and dendritic cells (Cole et al., 2005; He et al., 2009). Non-denaturing detergents enriched with mycoplasmal lipoproteins, have modulatory capacities (Cole et al., 2005; Liu et al., 2012). Synthetic lipopeptides such as the Mycoplasma-derived lipopeptide MALP-2 from *M. fermentans* (Muhlradt et al., 1997), FSL-1 (also known as Pam2C) from *M. salivarium* (Shibata et al., 2000), and MPPL-1 from *M. pneumoniae* (Into et al., 2007) were shown to have immunomodulatory properties. Activation of macrophages by mycoplasmal lipoproteins can occur at pico-molar concentrations of lipoproteins, making them extremely potent activators (Muhlradt et al., 1997).

*In vitro* and *in vivo* studies demonstrated that the *M. fermentans*-derived membrane component MALP-2 (macrophage-activating lipopeptide 2) is the main active component that induces these proinflammatory responses (Kaufmann et al., 1999). MALP-2 directly induced *in vitro* production of the proinflammatory cytokines TNF-α and IL-6 and the neutrophil-attracting CXC chemokines IL-8 and Growth-regulated Oncogene α (GRO-α) as well as the mononuclear leukocyte-attracting CC chemokines Monocyte chemoattractant protein-1 (MCP-1/CCL2), Macrophage inflammatory protein 1 alpha (MIP-1a/CCL3), and Macrophage inflammatory protein 1 beta (MIP-1b/CCL3) (Garcia et al., 1998; Rawadi et al., 1998). The MALP-2 molecule is a potent activator of human monocytes.

Mycoplasmal lipoproteins in *M. hominis* such as lipoprotein MHO_4720 were recently shown to induce production of Interleukin-23 (IL-23), by dendritic cells through activation of inflammasome (Chen et al., 2017; Goret et al., 2017). Production of proinflammatory cytokines such as IL-23 has been shown to induce the expression of chemokines such as IL-17, allowing for effective containment of the infection (Wu et al., 2007). Additional *in vivo* studies have shown that many of the chemokines induced by mycoplasma infection in humans are conserved across species, such as macrophage inflammatory protein (MIP)-1β (Lam, 2002). Certain mycoplasmal lipoproteins, such as those from *M. hyopneumoniae* also elicit anti-inflammatory cytokines such as Interleukin-10 (Chambaud et al., 1999; Mollazadeh et al., 2017).

Inducing apoptosis of both monocytes and lymphocytes is a key step in allowing Mycoplasma to prolong their survival within their hosts, and has been documented in numerous Mycoplasma, with *M. bovis* being one of the most well-studied (Into et al., 2004; Jimbo et al., 2017). Mycoplasma lipoproteins induce apoptosis in both monocyte (ex. HL-60, THP-1) and lymphocyte cell lines (ex. MOLT-4) (Aliprantis et al., 1999; Into et al., 2002a, 2004) and thus directly contribute to immune evasion.

Thus, mycoplasmal lipoproteins regulate myeloid based host immune responses and inflammatory responses, which are key mediators of pathogenesis of mycoplasmal infections. These innate immune responses may also drive responses from adaptive immunity (such as lymphocytic responses).

### Effects of Mycoplasmal Lipoproteins on Lymphocytes

Mycoplasmas induce proliferation of T and B cells (Naot et al., 1979; Cole et al., 1990) through superantigens or unidentified factors. Various mycoplasmas also activate cytotoxic T lymphocytes (CTLs) and natural killer (NK) cells (Wayner and Brooks, 1984) and induce expression of major histocompatibility antigens on B cells (Ross et al., 1992), IL-2 by T cells, interleukin (IL)-1 by macrophages (Muhlradt et al., 1991) and interferons (IFNs) by lymphocytes (Arai et al., 1983). Collectively these mycoplasma-driven immunomodulatory effects contribute to the proliferation and maturation of both T and B lymphocytes as well as the activation of macrophages and NK cells (Arango Duque and Descoteaux, 2014).

During the early phases of infection, mycoplasmas usually induce an inflammatory and a humoral response preferentially directed against their membrane-bound, surface-exposed lipoproteins. Mycoplasmal lipoproteins such as spiralin induce T-cell-independent B-cell blastogenesis and secretion of proinflammatory cytokines (Brenner et al., 1997). Lipoproteins from *M. fermentans* and *M. salivarium* induce TLR2- and caspase-mediated apoptosis in lymphocytes (Into et al., 2004). The effects of mycoplasmal lipoproteins on lymphocytes may not be mediated by effects on TLR signaling on antigen presenting cells such as dendritic cells (Sawahata et al., 2011). Thus, mycoplasmal lipoproteins also regulate adaptive immune and function of both B cells and T cells.

### Effects of Mycoplasmal Lipoproteins on Evasion of Host Immunity

Although lipoproteins have a major role as immune system activators, they also facilitate evasion of the host’s immune response, leading to chronic mycoplasma infections (Becker and Sander, 2016; Goret et al., 2016). Mycoplasma lipoproteins facilitate immune evasion through several mechanisms such as creation of the mycoplasmal shield, lipoprotein antigenic variation, and protection from growth inhibiting host antibodies (Citti et al., 1997; Chambaud et al., 1999). Understanding the various modes of immune system evasion promises a better approach to treating mycoplasma infections.

### Lipoproteins as the Basis for Mycoplasmal Shield Against Host Immune Defense Mechanisms

The ability of mycoplasma to encode numerous forms of lipoproteins that can potentially be expressed on their surface allows mycoplasmas to avoid host immune responses (Simmons et al., 2004). Mycoplasmas are able to create a protective layer around themselves that can sterically hinder access of growth-inhibiting host antibodies and even macrophages (Citti et al., 1997; Simmons et al., 2004). In addition to facilitating Mycoplasmal protection, the lipoprotein shield also plays an important role in hemadsorption and mycoplasmal adherence to surfaces (Bolland and Dybvig, 2012; Xiong et al., 2016). Mycoplasmas that are minimally shielded may be highly adherent while maximally shielded mycoplasmas are less adherent (Bolland and Dybvig, 2012).

### Variable Surface Antigen (Vsa) Lipoproteins as Facilitators of Immune Evasion

In addition to the generation of a shield for protecting Mycoplasmas, lipoproteins are also able to protect Mycoplasma by undergoing variation. One major method by which lipoprotein variation in mycoplasmas can contribute to host immune evasion involves altering the length of these variable surface antigens (Vsa) (Xiong et al., 2016). Mycoplasmas use antigenic variation not only to evade immune responses but also to adapt to each host (Bencina et al., 2001). Vsa lipoproteins normally have a 242-amino acid N-terminus with a variable C-terminal domain that can contain up to 60 tandem repeating units which in themselves can range in size from 10 to 19 amino acids (Bencina et al., 2001). Each cell can only transcribe one Vsa gene at a time, with silent Vsa genes missing the sequences necessary to make the conserved N-terminal domain (Shen et al., 2000). To achieve a large variety of Vsa recombination of lipoproteins, mycoplasmas use DNA recombination (Dybvig et al., 1998; Shen et al., 2000). Although a single cell may only transcribe a single Vsa gene, during an infection subpopulations of cells are able to express varying Vsa lipoproteins (Pantoja et al., 2016).

Despite the great importance Vsa size variation plays in facilitating prolonged mycoplasmal survival within hosts, adaptive immunity is still able to decrease mycoplasmal survival, thus necessitating phase variation of the shield as well (Simmons and Dybvig, 2009). Thus, in addition to being able to conduct size variation of surface lipoproteins, mycoplasmas are also able to conduct phase (type) variation of their lipoproteins, hence the name Vsa lipoproteins (Dybvig et al., 1998; Chambaud et al., 1999). Phase variation *in vivo* is only seen upon the onset of an adaptive immune response, with no alterations occurring in Vsa expression if an organism lacks B and T cells (Gumulak-Smith et al., 2001). It is estimated that phase switching occurs at a frequency of around 10^-3^ per CFU per generation (Rosengarten and Wise, 1990). In having the ability to undergo phase variation, mycoplasmas are able to evade host immune responses and indeed lead to chronic infections, similar to how Spirochetes might also use antigenic variation in order to establish a chronic infection (Chambaud et al., 1999; Christodoulides et al., 2017). Thus, Mycoplasmal lipoproteins have pleotropic immunomodulatory effects and their mechanisms of actions need to be elucidated.

## Mechanisms of Action of Mycoplasmal Lipoproteins

A key family of receptors that play an important role in recognizing, and inducing responses to Mycoplasmal lipoproteins are the Toll-Like Receptors (TLRs) that are key players in inflammatory responses upon infection (Akira et al., 2001; Wang et al., 2017). Two of the most well-studied TLRs are TLR2 and TLR6, both of which are pivotal in recognizing Mycoplasmal Macrophage-Activating Lipopeptide-2 (MALP-2), with the absence of either TLR leading to a lack of recognition and macrophage activation (Takeuchi et al., 2002). Mycoplasmal lipoproteins can also activate myeloid cells through the inflammasome (Chen et al., 2017; Goret et al., 2017). Amongst the Mycoplasma capable of activating inflammasome with their lipoproteins are *M. salivarium* and *M. pneumoniae* (Shimizu, 2016; Sugiyama et al., 2016). Through the activation of the inflammasome, the pre-cursors of proinflammatory cytokines, such as IL-1β and IL-18, can be cleaved and activated in a caspase-dependent manner prior to their release (Boyden and Dietrich, 2006).

Non-pathogenic Mycoplasma also express lipoproteins, suggesting that a non-TLR dependent, or inflammasome dependent, mechanism exists by which Mycoplasmal lipoproteins can lead to immune activation (Shimizu, 2016). *M. pneumoniae* was still able to induce a strong inflammatory response in TLR2 KO mice (Shimizu et al., 2014). Introduction of known autophagy inhibitors to the TLR2 KO mice in turn lead to a significant fall in the inflammatory response, suggesting a role of autophagy in also leading to inflammatory responses (Shimizu et al., 2014).

Another mechanism by which Mycoplasmal lipoproteins are able to lead to immune system activation is through their initial adhesion to epithelia such as the respiratory tract in the case of *M. pneumoniae* (Jordan et al., 2001). Such cytadherence is mediated through adhesins such as P1(MPN141) as well as other accessory proteins, and treatment of Mycoplasma with proteases or anti-P1 antibodies was seen to lead to a significant decrease in proinflammatory cytokine release (Yang et al., 2002; Hoek et al., 2005). Cytadherence on behalf of *M. pneumoniae* was seen to generate a proinflammatory response through pathways involving both autophagy/TLR4 as well as inflammasome (Shimizu et al., 2014).

In addition to their roles as inductors of the inflammatory response, TLRs also play an important role in inducing apoptosis in both monocyte cell lines (ex. HL-60, HEK293, THP-1) and lymphocyte cell lines (ex. MOLT-4) upon binding of Mycoplasmal lipoproteins (Aliprantis et al., 1999; Into et al., 2002a; Into et al., 2004). The two main TLRs seen to play a role in this apoptotic pathway are TLR-2 and TLR-6, which through their activation allow induction of NF-kB (Aliprantis et al., 1999; Wu et al., 2008) and the release of Nitric Oxide from macrophages (Muhlradt and Frisch, 1994; Into et al., 2004). Mycoplasmal induced apoptosis seems to be mediated by activation of the p38 MAPK, the apoptosis signal-regulating kinase (ASK1) and the NF-kB pathways (Into and Shibata, 2005). Mycoplasmas such as *M. salivarium* and *M. fermentans* have also been seen to induce apoptosis through the more conventional caspase pathways (Into et al., 2002a,b).

On the other hand, certain mycoplasmal lipoproteins, such as those seen in *M. fermentans*, can inhibit TNF-α mediated apoptosis (Gerlic et al., 2007). Thus, further research is needed to understand the pleotropic effects of mycoplasmal lipoproteins on apoptosis.

A better understanding of mechanisms of immunomodulation mediated by mycoplasmal lipoproteins may lead to therapies to attenuate the detrimental effects of mycoplasma infections.

## Mycoplasmal Lipoproteins as Therapeutic Targets

One of the most difficult parts of managing a Mycoplasmal infection is the inability to use many antibiotics such as beta-lactams (Bebear and Pereyre, 2005). The development of drugs such as tigecycline that aim to reduce production of chemokines and lipoproteins through targeting of bacterial ribosomes is a growing discipline within the field of Mycoplasma research (Saliba et al., 2009; Salvatore et al., 2009). Studies of tigecycline in a murine model have been able to confirm a significant drop of cytokine and chemokine production following infection with *M. pneumoniae* (Salvatore et al., 2009). Many studies that focus on development of a possible vaccine against Mycoplasma use inactivated Mycoplasma that can then be introduced to patients (Wenzel et al., 1977; David et al., 2010). Atalla et al. (2015) were able to show that inactivation of Mycoplasma using a photoactivatable alkylating agent (INA), with preservation of the surface lipoproteins, might be a possible mechanism by which Mycoplasma can be inactivated for vaccine development (Atalla et al., 2015). Through a better understanding of bacterial lipoproteins and their interaction with the immune system, researchers are now aiming to develop vaccines that rely on the introduction of bacterial lipoproteins into patients to generate antibody responses (Leng et al., 2015). For example, the above has been achieved with the AvgC lipoprotein from *M. agalactiae* and shows promise for a vaccine against *M. agalactiae* infection (Santona et al., 2002). Such an approach to vaccine development is also currently being studied using bacterial lipoproteins, such as the OspA from Spirochetes (Izac et al., 2017; Zhao et al., 2017). An innovative approach for treatment of Mycoplasmal infections would involve direct targeting of lipoproteins; however, such an approach remains to be established given the structure of mycoplasmal lipoproteins and their diverse immunomodulatory mechanisms. Importantly, the polypeptide Aspidasept^®^ which neutralizes endotoxin *in vivo* was previously shown to inhibit cytokines induced by the Mycoplasmal lipoproteins (Martinez de Tejada et al., 2015). Thus, a better understanding of Mycoplasmal lipoprotein epitopes, may promote progress in the field of antibodies, polypeptides or vaccines against Mycoplasma.

## Conclusion

Further studies are needed to promote understanding of the mechanisms of transcriptional regulation, phase variations and the numerous immunomodulatory effects (both acute and chronic) of Mycoplasmal lipoproteins. Given the decreasing efficacy of antibiotics against mycoplasmas, there is need for novel immunotherapies. Novel strategies such as use of inactivating agents that preserve the mycoplasmal surface lipoproteins, may advance vaccine development (Atalla et al., 2015). Novel approaches such as use of polypeptides that attenuate proinflammatory effects of mycoplasmal lipoproteins need further investigation as novel therapeutic approaches for mycoplasmal infections (Martinez de Tejada et al., 2015). Ultimately, a better understanding of mycoplasmal lipoproteins can set the basis for the development of vaccines or antibodies against mycoplasmal lipoproteins and perhaps shed light on the pathogenesis of other vector based pathogens such as Spirochetes.

## Author Contributions

AC, NG, VY, JP, NM, and TK wrote and edited the manuscript. TK conceptualized the manuscript.

## Conflict of Interest Statement

The authors declare that the research was conducted in the absence of any commercial or financial relationships that could be construed as a potential conflict of interest.
